# Learning intrinsic excitability in medium spiny neurons

**DOI:** 10.12688/f1000research.2-88.v2

**Published:** 2014-02-26

**Authors:** Gabriele Scheler

**Affiliations:** 1 Carl Correns Foundation for Mathematical Biology, Mountain View, CA, 94040, USA

## Abstract

We present an unsupervised, local activation-dependent learning rule for intrinsic plasticity (IP) which affects the composition of ion channel conductances for single neurons in a use-dependent way. We use a single-compartment conductance-based model for medium spiny striatal neurons in order to show the effects of parameterization of individual ion channels on the neuronal membrane potential-curent relationship (activation function). We show that parameter changes within the physiological ranges are sufficient to create an ensemble of neurons with significantly different activation functions. We emphasize that the effects of intrinsic neuronal modulation on spiking behavior require a distributed mode of synaptic input and can be eliminated by strongly correlated input. We show how modulation and adaptivity in ion channel conductances can be utilized to store patterns without an additional contribution by synaptic plasticity (SP). The adaptation of the spike response may result in either "positive" or "negative" pattern learning. However, read-out of stored information depends on a distributed pattern of synaptic activity to let intrinsic modulation determine spike response. We briefly discuss the implications of this conditional memory on learning and addiction.

## Introduction

A role for modification of activation functions, or intrinsic plasticity (IP), for behavioral learning has been demonstrated for a number of systems ^1– 3^. For instance, in rabbit eyeblink conditioning, when ion channels related to after hyperpolarization are being suppressed by a learning event, they can become permanently suppressed. This has been shown for pyramidal cells of hippocampal areas CA1 and CA3, and for cerebellar Purkinje cells ^4, 5^. In some cases, these changes are permanent and still present after 30 days ^6, 7^, in other cases, intrinsic changes disappear after 3–7 days, while the behavioral memory remains intact, raising questions about the long-term component of intrinsic plasticity in these systems. There are at the present time conflicting ideas on the significance of IP compared to synaptic plasticity ^1, 8^, and the range of functions that IP may have in adaptivity ^9– 12^.

A few computational models have been proposed that show how modification in activation functions can be achieved with ion channel based models of realistic single neurons. Marder and colleagues have developed an approach, where they sample a very large parameter space for conductances of ion channels, exploring nonlinearities in the relation between conductances and neural spiking behavior ^13– 15^. The motivation for this research are observations about neuromodulation and intrinsic plasticity in specific neurons of an invertebrate ganglion (e.g. LeMasson *et al.*, ^16^). They have noted that large variations in some parameters may have little effect on neuronal behavior, while comparatively small variations in certain regions in parameter space may change response properties significantly. They also suggest that neuro modulation may provide an efficient means of targeting regions in parameter space with significant effects on response properties ^14^.

A study by Stemmler and Koch ^17^ assumed the goal of modification of activation functions is to achieve an optimal distribution of firing rates for a population of neurons. The idea was that by tuning each neuron to a different band of the frequency spectrum, the full bandwidth of frequencies could be employed for information transfer. This goal was achieved by adjusting *Na* ^+^, *K* ^+^, and *Ca* ^++^ channels for a generically defined neuron until a desired frequency was stably reached.

We present a different approach, where the modification of activation functions reflects the history of exposure to stimuli for a specific neuron. In previous work ^18, 19^, it was suggested that synaptic LTP/LTD and linear regulations of intrinsic excitability could operate in a synergistic fashion. However, in our approach, different types of synaptic stimulation result in state changes for the neuronal unit, influencing its capacity for read-out of stored intrinsic properties. Different types of synaptic stimulation result in changes of neural transmission properties — synchronized input leading to a timed spike regime and distributed input leading to a ‘read-out’ of the intrinsic frequency or gain function. Thus, intrinsic plasticity, in contrast to synaptic plasticity, is only expressed or ‘read out’ under conditions of ongoing background stimulation, not in the presence of strong synchronous input. The learning rule that we derive as the basis for adjustment concerns one-dimensional upregulation or down-regulation of excitability in the “read-out” state of the neuron, and affecting only this state. This rule uses neural activation, significantly determined by intracellular calcium for the learning parameter, which can be shown to be biologically well-motivated (cf. also e.g. LeMasson *et al.*, ^16^).

## Materials and methods

### Striatal medium spiny neuron

The membrane voltage *V _m_* is modeled as

$${\dot{V}}_{m}=-\frac{1}{C}\left[\sum_{i}I_{i}-I_{syn}\right]$$

The individual currents are modeled by conductances, state variables and the reversal potential:

$$I_{i}={\bar{g}}_{i}\left(V_{m}\right)*m^{pi}*h_{i}^{qi}*(V_{m}-E_{i}^{rev}) (1)$$

The dynamics are defined using state variables for activation ( *m*) and inactivation ( *h*). The types of equations used for the dynamics are:

    1. exponential: $f\left(V_{m}\right)=\lambda \exp\left(\frac{V_{m}-V_{i}}{-V_{c}}\right)$

    2. logistic: $f\left(V_{m}\right)=\frac{\lambda }{1+\exp\left(\frac{V_{m}-V_{i}}{-V_{c}}\right)}$

    3. linexp: $f\left(V_{m}\right)=\frac{\lambda \left(V_{m}-V_{i}\right)}{1+\exp\left(\frac{V_{m}-V_{i}}{-V_{c}}\right)}$

The state variables can be defined indirectly using

$$\dot{m}=(1-m)\;\alpha -m\beta$$

and

$$\dot{h}=(1-h)\;\alpha -h\beta$$

and one of the ( Equation 1– Equation 3) with different values for λ (λ _*α*_, λ _*β*_), *V _i_* ( *V _i_* ^*α*^, *V _i_* ^*β*^) and *V _c_* ( *V _c_* ^*α*^, *V _c_* ^*β*^). We use this method for the ion channels in Table 1.

**Table 1.** Parameter values for *I _Na_, I _K_, I _leak_* as in ^20^, *I _CaL_* as in ^21, 22^ for activation (m) and inactivation (h) (see Equation 1). Exponents (p, q), conductance $\bar{g}$ (in mS) and parameters are shown. The types of equations ( *Eq ^α^, Eq ^β^*) are 1 for exponential, 2 for logistic, and 3 for linexp (see text for the equations). The reversal potential *E ^rev^* is given in mV.

I	p	q	$\bar{g}$	λ _*α*_	*V _c_* ^*α*^	*V _i_* ^*α*^	*Eq ^α^*	λ _*β*_	*V _c_^β^*	*V _i_* ^*β*^	*Eq ^β^*	*E ^rev^*
Na (m)	3		35	0.1	10	–28	3	4.0	18	–53	1	55
Na (h)		1		0.07	20	–51	1	1	10	–21	2	
K	4		6	0.01	10	–34	3	0.125	80	–44	1	–90
CaL (m)	2		0.01	0.06	3.8	–40	3	0.94	17	–88	1	140
CaL (h)		1		4.6e-4	50	–26	1	6.5e-3	28	–28	2	
leak			0.04									–75

The state variables can also be directly defined (cf. Goldman *et al.*, ^14^):

$$\begin{array}{l} \dot{m}=\frac{m_{\infty }-m}{\tau_{m}}\\ \dot{h}=\frac{h_{\infty }-h}{\tau_{h}} \end{array}$$

The parameters used are *m*_∞_ = *m*0, *h*_∞_ = *h*0, τ _*m*_ and τ _*h*_ as in Table 2. Again, we use one of the ( Equation 1– Equation 3) with the λ parameters (λ _*m*0_ and λ _*h*0_) set to 1. These representations are mathematically equivalent and related by

$$m_{\infty }=\frac{\alpha_{m}}{\alpha_{m}+\beta_{m}},\;\tau_{\infty }=\frac{1}{\alpha_{m}+\beta_{m}}$$

**Table 2.** Parameter values for potassium channels *I _Kir_*, *I _Af_*, *I _As_* and a slow sodium channel *I _Nas_* cf. ^27, 28^, where (a) τ *_m_* = 131.4/(exp(–( *V _m_* + 37.4)/27.3) + exp(( *V _m_* + 37.4)/27.3)) (b) τ *_h_* = 179.0 + 293.0 * *exp*(-(( *V _m_* + 38.2)/28) ^2^) * (( *V _m_* + 38.2)/28) (c) τ *_m_* = 637.8/(exp(–( *V _m_* + 33.5)/26.3)+exp (( *V _m_* + 33.5)/26.3)). Exponents (p, q), conductance $\bar{g}$ (in mS), parameters *m*0 and *h*0 for a logistic function (equation type 2), and time constants τ *_m_*, τ *_h_* are shown. The reversal potential *E ^rev^* is given in mV.

I	p	q	$\bar{g}$	*V _c_* ^*m*0^	*V _i_* ^*m*0^	*Eq* ^*m*0^	*V _c_* ^*h*0^	*V _i_* ^*h*0^	*Eq ^h0^*	τ *_m_*	τ *_h_*	*E ^rev^*
Kir	1		0.15	–10	–100	2				<0.01	<0.01	–90
Af	1	1	0.09	7.5	–33	2	–7.6	–70	2	1	25	–73
As	1	1	0.32	13.3	–25.6	2	–10.4	–78.8	2	(a)	(b)	–85
Nas	1		0.11	9.4	–16.0	2				(c)	<0.01	40

Standard parameter values for the modeling of ion channels (“naive state”) were compared with several publications. Parameter values for *I _K_*, *I _Na_* and *I _leak_* were adapted from ^20^, for L-type calcium channels ( *I _CaL_*) from ^21^ and ^22^, see Table 1.

Parameters for slow A-type K channels ( *I _As_*) were adapted from ^23, 24^, for fast A-type K channels ( *I _Af_*) from ^25^, for inward rectifying K channels ( *I _Kir_*) from ^26^, and the resulting parameter tables were compared with ^27^ and ^28^, see Table 2.

### Modulation

The maximum conductance of different ion channels can be expressed by a scaling factor in the membrane potential equations as in Eq. 2 (for synaptic currents *I _i_*) or Eq. 3 (for synaptic conductances *g _s_*), cf. Gruber *et al.*, ^28^.

$${\dot{V}}_{m}=-\frac{1}{C}[\mu_{1}I_{1}+\mu_{2}I_{2}\ldots +\mu_{i}I_{i}-I_{syn}] (2)$$

$${\dot{V}}_{m}=-\frac{1}{C}[\mu_{1}g_{1}(V_{m}–V_{0})+\ldots +g_{s}\left(V_{m}-V_{0}\right)] (3)$$

Both neuromodulator (NM)-independent and NM-dependent modifications may coexist in a neuron, as expressed in Eq. 4 ([NM] stands for the level of synaptic availability of a neuromodulator NM).

$${\dot{V}}_{m}=-\frac{1}{C}[\left(\mu_{1}I_{1}+\left[NM\right]\kappa_{1}I_{1}\right)+\left(\mu_{2}I_{2}+\left[NM\right]\kappa_{2}I_{2}\right)\ldots ] (4)$$

In this paper, for simplicity, we shall refer to ( Eq. 2) as the generic format for intrinsic adaptation, with the understanding that *μ* is replaceable by [ *NM*] *κ*.

Physiological ranges for *μ* can be estimated by various means. There are measurements for modulation in electrophysiologically defined membrane behavior (current threshold, spike response to current pulses etc. ^29, 30^) that are typically expressed as standard errors (e.g., 16–20% for current threshold ^29^). There are also attempts at classifying MSN (Medium Spiny Neuron) cells into different ‘types’ based on their electrophysiological profile ^31, 32^. Modeling shows that modulation of ion channel conductances with a range of ±40% matches measures of electrophysiological modulation and reproduces the ranges for MSN types (data not shown). Interestingly, direct measurements for dopamine D1 receptor-mediated changes on ion channel conductances are approximately in the same ranges (±30–40% ^28^). Our discussion is thus based on an estimate of *μ* ranging from 0.6–1.4 for each channel.

### Defining synaptic input

Synaptic input is defined by overlays of the excitatory postsynaptic potentials (EPSPs) generated by *N* individual Poisson-distributed spike trains with mean interspike interval τ _*syn*_. Each EPSP is modeled as a spike with peak amplitude *I*_0_ = 1.2 *μ*A/cm ^2^ and exponential decay with τ = 2.5ms similar to ^22, 33^. IPSPs are modeled in a similar way with *I*_0_ = –0.4 *μ*A/cm ^2^. This corresponds to 0.5nA (–0.2nA) as peak current (with 1nA = 2.3 *μ*A/cm ^2^). Synaptic conductances are calculated by *g*_*syn*_ = *I*_*syn*_/( *V*_*m*_ – *V*_0_) with *V*_0_ set to 0mV. In order to be consistent with our simulation environment we use the units *μA/cm* ^2^ and *mS/cm* ^2^ to describe the spikes, rather than a voltage. We have tuned the model to *g*_*syn*_= 0.0035mS/cm ^2^ to induce a first spike for the naive or standard neuron (all *μ* = 1). At –40mV (firing threshold), this is 0.0035mS/cm ^2^ * (–40mV) = – 1.4 *μ*A/cm ^2^ or 0.6nA, which corresponds to the experimentally measured average value for the rheobase from resting potential in ^29^. We may increase the correlation in the input by using a percentage *W* of neurons which fire at the same time. Higher values for *W* increase the amplitude of the fluctuations of the input (cf. Benucci *et al.*, ^34^). For details see the Matlab implementation.

### Implementation

The simulator has been implemented in Matlab and is available at https://github.com/gscheler/CNeuroSim.git. The entire code is interpreted and no specific code optimizations have been applied. For numerical integration, the solver ode45 was used.

## Results

### Intrinsic modulation

**Figure 1.**
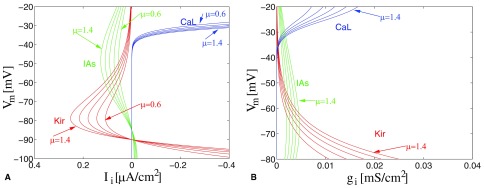
Modulation of ion channel density. Variable factors ( *μ* = {0.6 ... 1.4}) for the slowly inactivating *K* ^+^-channel (Kv1.2, *I _As_*), the L-type calcium channel ( *I _CaL_*), and the inward rectifying *K* ^+^ channel ( *I _Kir_*) are shown at different membrane voltages *V _m_* ( **A**) in an I-V plot, ( **B**) as modulation in conductance.

**Figure 2.**
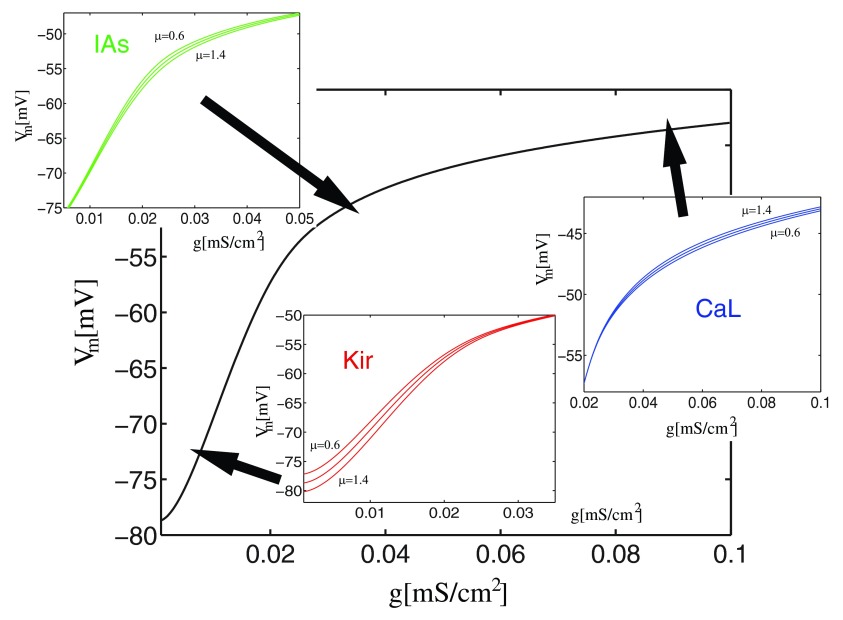
Modulation of the activation function. Variable factors ( *μ* = {0.6 ... 1.4}) for *I _As_*, *I _CaL_*, and *I _Kir_* as components of the activation function ( *g _s_* vs. *V _m_*). This function is defined as the membrane voltage response for different injected (synaptic) conductances ( *g _s_*), and computed by solving Eq 3 for the membrane voltage *V _m_*.

We explore the impact of small variations in ion channel conductances on the shape of the activation function. As an example, we show the current and conductance changes for a slowly inactivating A-type *K* ^+^ channel (Kv1.2, *I _As_*), L-type calcium channel ( *I _CaL_*) and inward rectifying *K* ^+^ channel ( *I _Kir_*) at different membrane potentials modulated by a scaling factor *μ* = {0.6, 0.8, 1.0, 1.2, 1.4} ( Figure 1, Figure 2). Regulation of the voltage-dependence ^35^ and even of the inactivation dynamics of an ion channel ^36^ has also been shown, but these effects are not further discussed here.

We can see that there are critical voltage ranges (around –50mV, around –80mV and starting at –40mV), where the conductance and the current are highest, and where scaling has a significant effect, while scaling has small or no effect in other voltage ranges. (The *Na* ^+^ current has been disabled for this example to prevent the neuron from firing).

**Figure 3.**
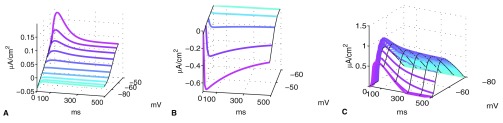
Activation-inactivation (temporal) dynamics. ( **A**) dynamics for the slow A channel *I _As_* ( **B**) the L-type Ca channel *I _CaL_*, and ( **C**) for the set of ion channels used in the standard MSN model. We see a rise-time due to *I _As_* and overlapping inactivation dynamics in the -55 to -40 mV range.

In Figure 3, we show the current over time–to graphically display the slow dynamics of the *I _As_* and *I _CaL_* channel. Since we do not change the activation-inactivation dynamics of any channel in our model, we show currents only for *μ _As_*, *μ _CaL_* = 1.

We can see that *I _As_* activates moderately fast (20ms), while it inactivates with a half-time of about 300ms, depending on the voltage. For *I _CaL_*, activation is almost instantaneous, but inactivation is > 500ms.

The activation function for the MSN model shows a time-dependence only in the high-voltage range (at or above –55mV), whereas the components in the lower voltage ranges are not time-dependent.

Mathematically, we can consider the individual channels as a set of functions that allow function approximation for the activation function. Each particular adjustment of an activation function can be considered learning of a filter which is suited to a specific processing task. The activation-inactivation dynamics would provide a similar set of functions for the temporal domain. Of course, it is interesting to note which particular basis functions exist, and also how the temporal dimension is tied in with specific voltage-dependences. For instance, the slowly inactivating potassium channel *I _As_* provides a skewed mirror image of the function of calcium-gated Sk/BK channels, which are responsible for after hyperpolarization, making different variants of frequency filters possible. On this basis, a mapping of ion channel components and their density or distributions in different types of neurons could provide an interesting perspective on direct interactions for neurons from different tissue types or brain areas, as well as e.g., between cholinergic interneurons and MSNs within striatum.

**Figure 4.**
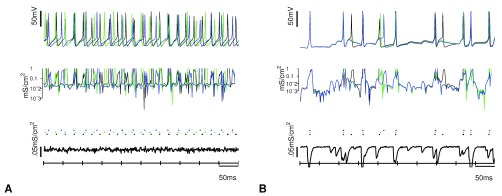
Input correlation-dependent read-out of intrinsic memory. Response to inputs generated from *N* = 80 neurons firing with 20Hz with independent Poisson processes using different correlation parameters *W* = 0.2, 0.9 ( **A**, **B**). Extreme values of correlations have been chosen for demonstration purposes. Three slightly different neurons with *μ _As_* = 1.1,1.3,1.5 are shown under BOTH conditions. ( **A**) Response modulation and different firing rates for each neuron (here: 20, 26, 40Hz) occur with distributed (low correlation) input. ( **B**) Highly correlated input produces reliable spiking and by implication a single firing rate (20Hz). The upper panel shows the membrane voltage, the middle panel shows the membrane conductances, and the lower panel shows the synaptic input as conductance.

To further explore the influence of modulation on the activation function, we apply realistic synaptic input with different amounts of correlation to individual MSNs (see Figure 4).

This shows us that small adjustments in the contribution of a specific ion channel can result in significantly different spiking behavior even for identical synaptic input. This occurs when the input is distributed, i.e., has low correlation. In this case, the neurons spike independently of each other and with different frequencies. We can eliminate this effect by increasing the correlation of the input. Because of the slow activation/inactivation dynamics of the *I _As_* channel, (latency of ≈ 20ms) only low correlated input activates these channels (“neuronal integrator mode”), but highly correlated inputs do not activate these channels, driving the membrane to spiking quickly (“coincidence detector mode”). Therefore correlated input can produce reliable spiking behavior for model neurons which differ in the relative contribution of the slow *I _As_* channel. Distributed input, in contrast, activates slower ion channels, and can produce different tonic firing rates, here according to the contribution of the *I _As_* channels, as long as strong synaptic input keeps the neuron in the relevant voltage range (“persistent activity”).

Similarly, the differential contribution of other channels (high-voltage gated L-type Ca-channels, hyperpolarization-activated GIRK channels or calcium-dependent Sk/Bk channels) will affect neuronal behavior, when the conditions for a prominent influence of these channels are met.

We are modeling a state of MSNs that exhibits regular tonic firing. In experimental studies ^37^, showed that MSNs, similar to cortical neurons, exhibit upstate-downstate behavior, reminiscent of slow-wave sleep, under certain forms of anesthesia (ketamine, barbiturate). However, under neuroleptanalgesia (fentanyl-haloperidol) ^50^, showed that MSNs can show driven activity, when cortical input is highly synchronized, and exhibit a state characterized by fluctuating synaptic inputs without rhythmic activity (i.e., without upstates/downstates), when cortical input is desynchronized. The regular, tonic spiking in this state is very low, much less than in the waking animal, which may be related to the dopamine block by haloperidol. This makes a waking state of MSNs characterized by regular tonic spiking at different firing rates probable.

In the following, we show how intrinsic excitability adaptation can lead to different recalled firing rates under appropriate synaptic stimulation. The model could thus reflect learning that is recalled or read out during MSN states under desynchronized cortical input–in contrast to highly synchronized input, which would homogenize the response of the coincidence detecting neurons and favor reliable transfer of spikes.

### Induction and maintenance of plasticity

The general idea for learning intrinsic plasticity is to use a learning parameter *h* for each individual update of the conductance scaling factor *μ*. The *direction* of learning ( *h* > 0 or *h* < 0) is determined from the neural activation ( *A _n_*) for each individual neuron. Neural activation is largely determined by intracellular calcium. We calculate the neural activation *A _n_* from the spike rate of the neuron, measured over 1s of simulated behavior (see Discussion).

We define a bidirectional learning rule dependent on an initial firing rate *θ*: excitability is increased by a step function *h* (with stepsize *σ*) when *A _n_* is greater than *θ*, excitability is decreased when *A _n_* is lower than *θ* (“positive learning”). This means, when the actual neural activation is higher than the initial firing rate, membrane adaptations aim to move the neuron to a higher excitability in order to create a positive memory trace of a period of high activation (which can then be replicated under distributed synaptic stimulation). The same mechanism applies to lower the excitability of a neuron.

Δμ=h(An)=σ if An>θΔμ=h(An)=−σ if An<θ  (5)

This rule can also be implemented by individual increases in excitability after each action potential, and decreases of excitability for periods of time without action potentials. Experimental data ^38, 50^ indeed show such adaptation of intrinsic excitability after individual spikes.

The function *h* can be applied to a single ion channel, such as *I _As_*, but also to a number of ion channels in parallel: e.g., to mimic dopamine D1 receptor activation, *h* may be applied to *μ _As_* (upregulated with high *A _n_*), *μ _Na_* (downregulated with high *A _n_*), and *μ _CaL_* (downregulated with high *A _n_*). We suggest that there exists a biological feedback mechanism between the firing rate *θ* and the regulation of ion channel density and expression via intracellular signaling pathways, probably mediated by intracellular calcium level.

### Pattern learning

**Figure 5.**
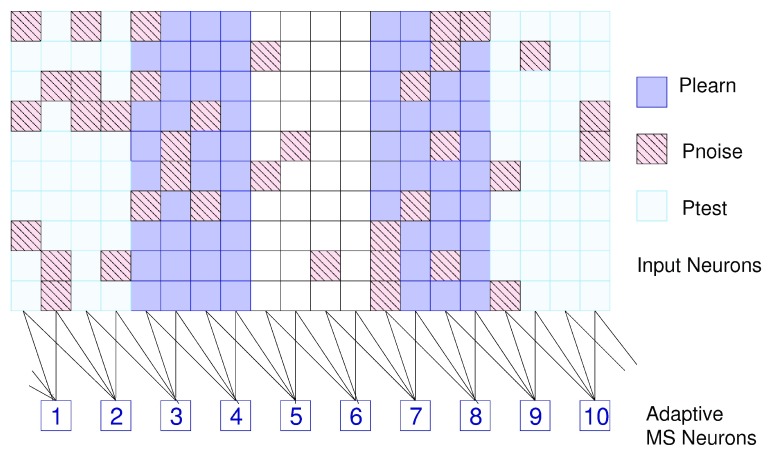
Pattern learning. 200 input neurons (arranged as 20 × 10), 10 learning neurons, and definitions for 3 patterns. Only 37 of 80 data points for *P _noise_* are shown.

We can show the effect of this learning rule on pattern learning. We generate synaptic inputs from a grid of 200 input neurons for a single layer of 10 MSNs. On this grid we project two stripes of width 4 as a simple input pattern *P _learn_* by adjusting the mean interspike interval (ISI) for the corresponding input neurons to a higher value *(ISI* = 350ms for *on* vs. 750ms for *off* neurons, see Figure 5).

We apply the learning rule to each of the currents *I _Na_, I _CaL_* and *I _As_*. This mimics changes in dopamine D1 receptor sensitivity, which targets these ion channels. Adaptation can be weaker or stronger, depending on learning time (e.g., σ = 0.01, *t* = 20 *s* (20 steps) (weak), *t* = 40 *s* (40 steps) (strong)). After a number of steps, we achieve a distribution of *μ*-values that reflects the strength of the input ( Table 3A).

**Table 3.** Pattern learning: Parameter values (A) for positive learning with (weak/strong) adaptation of *μ* values and (B) negative learning. CaL and Na channels are adapted in the opposite direction to K channels.

no	*μ _CaL_*	*μ _As_*	*μ _Na_*						no	*μ _CaL_*	*μ _As_*	*μ _Na_*
1	0.9/0.8	1.2/1.4	0.9/0.8						1	1.4	0.7	1.0
2	0.9/0.8	1.2/1.4	0.9/0.8						2	1.4	0.7	1.1
3	1.0	1.0	1.0						3	0.9	1.1	1.0
4	1.1/1.2	0.8/0.6	1.1/1.2						4	0.6	1.4	0.8
5	1.0	1.0	1.0						5	1.1	0.8	0.9
6	0.9/0.8	1.2/1.4	0.9/0.8						6	1.4	0.7	1.1
7	1.0	1.0	1.0						7	1.1	0.8	0.9
8	0.9/0.8	1.2/1.4	0.9/0.8						8	0.7	1.4	0.8
9	1.0	1.0	1.0						9	1.0	0.9	0.9
10	0.9/0.8	1.2/1.4	0.9/0.8						10	1.4	0.7	1.1
**A**									**B**			

**Figure 6.**
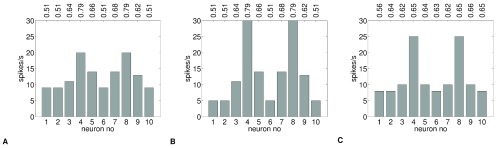
Positive pattern learning. Spike frequency histograms for 10 adaptive neurons ( *θ*=11.5Hz) ( **A**) response of naive neurons to *P _learn_* ( **B**) response of *P _learn_*-adapted neurons to *P _learn_* ( **C**) *P _learn_*-adapted neurons tested with *P _noise_*. Average synaptic input (nA/cm ^2^) for each neuron is shown on top. Responses in ( **A**) and ( **B**) to the same input *P _learn_* are different, a pattern similar to *P _learn_* emerges in response to uniform (noise) pattern input in ( **C**).

In Figure 6, we obtain spike frequency histograms from the set of MSNs under different conditions. Figure 6A shows the naive response to the input pattern *P _learn_*–high activation in two medial areas. After adaptation, this response is increased ( Figure 6B). When we apply a test input of a random noise pattern *P _noise_*, we see that the learned pattern is still reflected in the spike histogram ( Figure 6C). This retention of pattern learning in the presence of noise could be further explored with a detailed statistical analysis while quantifying noise levels. For positive learning, this process is theoretically unbounded, and only limited by the stepsize and the adaptation time. A saturation state could be defined to prevent unbounded learning, which would also allow to perform capacity calculations.

We should note that applying just one pattern continuously results in a very simple learning trajectory: each update results in a step change in the relevant ion channel currents. However, we also show that the effects of stepwise adaptation of individual ion channels do not necessarily lead to a completely parallel adaptation of firing rate. In Figure 6 we see that adaptation is much stronger for high input rather than low input neurons. In this case, *θ* at 11.5Hz is a fairly low value for neurons to continue to lower their firing rate with stepwise adaptation of the chosen ion channels. This shows the importance of using appropriate tuning (“harnessing”) mechanisms to make highly nonlinear channels work in a purely linear learning context.

Clearly one of the results of learning is an altered spiking behavior of individual neurons dependent on their history. It is important to realize that this rule is based on neural activation, not synaptic input as a learning parameter - since synaptic input is constant during learning.

### Positive and negative trace learning

**Figure 7.**
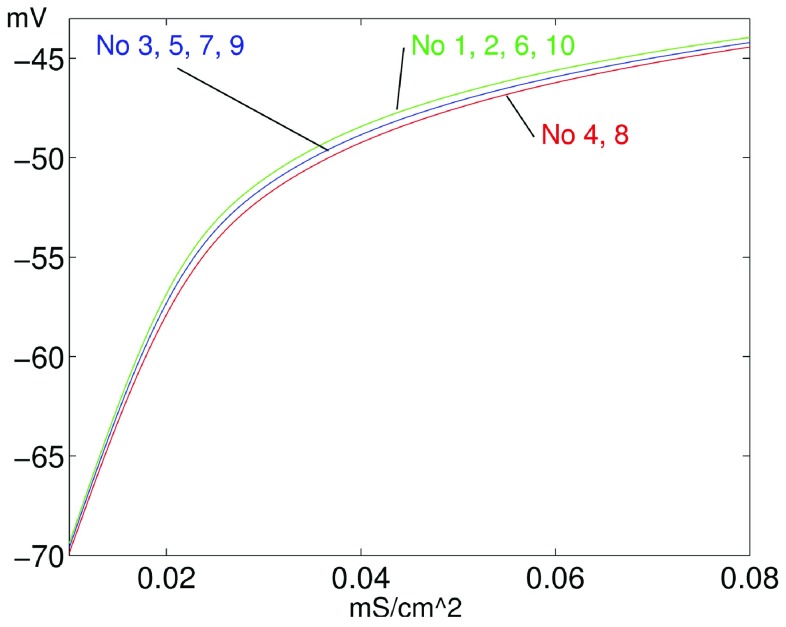
Negative pattern learning. Learning results in different activation functions for high (4, 8), medium (3, 5, 7, 9) and low (1, 2, 6, 10) input.

We show that this mechanism can be employed not only for positive trace learning, when excitability adaptation corresponds to frequency response, but also for negative trace learning, when excitability adaptation counteracts frequency response and approximates a target firing rate *θ*. This target rate could be set as a result of global inhibitory mechanisms corresponding to the expected mean *A _i_* values under physiological stimulation. Accordingly, the neuron responds with decreases of excitability to high input ranges and increases of excitability to low input ranges ( Figure 7).

Δμ=h(An)=−σ if An>θΔμ=h(An)=σ if An<θ  (6)

This emphasizes that “homeostatic” responses - adjusting excitability in the opposite direction to the level of input - can implement trace learning (pattern learning and feature extraction) as well. We note that this learning rule is not guaranteed to be stable beyond our own simulation runs. Additional mechanisms, such as concurrent synaptic plasticity, may be necessary to provide a stable learning regime.

**Figure 8.**
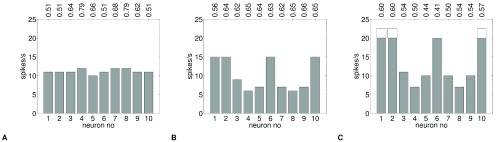
Negative pattern learning. Spike frequency histograms for 10 adaptive neurons ( *θ*=11.5Hz) ( **A**) habituation for neurons adapted to *P _learn_*, ( **B**) an inverse pattern for *P _learn_*-adapted neurons tested with *P _noise_* and ( **C**) interference (dampening of response) for a new pattern *P _test_* (naive: line-drawn bars, adaptive: filled bars). Average synaptic input (nA/cm ^2^) for each neuron is shown on top. ( **A**) shows a uniform response to patterend synaptic input and ( **B**) a patterned response to uniform (noise) input. ( **C**) shows a difference of response for naive vs. *P _learn_*-adapted neurons to a new pattern *P _test_*.

The negative learning rule results in a mirror image of parameter values compared to positive learning, as shown in Table 3B. The naive response is the same as before ( Figure 6A). But here, after adaptation, the neurons have habituated to the input, and do not produce a strong response anymore ( Figure 8A). When neurons are tested with *P _noise_*, an inverse version of the original pattern appears ( Figure 8B). Similarly, when we apply a different pattern *P _test_*, we obtain a spike histogram, where the learned pattern is overlayed with the new input, resulting in a dampening of the frequency response for *P _test_* ( Figure 8C).

In our simulations we have applied either a positive or a negative learning rule. It is an open, and very interesting question, whether both types of learning may occur in a single tissue, distributed over different neurons, or neuron types, or whether only one of these forms will prove to occur in a real biological context.

For both positive and negative traces, learning is pattern-specific, i.e., training with homogeneous, fluctuating (high-low) noise, such as *P _noise_*, results in no adjustments (or computes an average). However, any prolonged sequence of neuron-selective stimulation results in neuron-selective patterns. This requires the population to be protected from prolonged stimulation with random patterns in a biological setting. We may assume most patterns to be meaningful and highly repetitive, while the neuron exists in a plastic state, while patterns may be random, when the neuron is not plastic (because it is stimulated with highly correlated or very low frequency input, saturated in its parameters or undergoes ion channel block by selected neuromodulators).

The whole approach to pattern storage and responses elicited to stimulation is summarized again in Figure 6 and Figure 8. We can see that pattern storage by changes in intrinsic excitability is useful for a short-term buffer system for complete patterns. Patterns are imprinted upon a set of neurons and remain available as long as they are not obliterated or overwritten by an opposite pattern. Presumably the pattern degrades over time. Training with a new pattern – during the period of active maintenance of the pattern–would result in cross-activation, i.e., the generation of a mixed pattern. This may well be a useful feature of a short-term pattern storage system. It allows for pattern integration, or pattern completion from different sources. Adapting intrinsic excitability has inherent limitations of storage capacity. We do not fully understand where patterns go after they have passed through the intrinsic buffer system, but we assume that synaptic growth, intracellular changes and membrane adaptations in a variety of trafficking proteins (receptors and channels) all play a role. In the simplest case, the intrinsic buffer system serves only to integrate and maintain a pattern of neural excitation until all the necessary synaptic adjustments that the memory system requires for permanent storage have been made. However, it is not clear, and actually highly doubtful at this time that the difference between short-term and long-term storage is clear-cut between intrinsic (neuronal) and synaptic (esp. glutamatergic synaptic) storage systems.

## Discussion

### Experimental results on induction of intrinsic plasticity

A number of experimental results show that intrinsic plasticity in MSNs may be prominently induced and regulated by intracellular calcium: It has been shown that e.g., the regulation of delayed rectifier *K* ^+^-channels (Kv2.1 channels) is effectively performed by *Ca* ^2+^ influx and calcineurin activation in cultured hippocampal neurons, which can be achieved by glutamate stimulation ^35^. The regulation concerns marked dephosphorylation (reduction of conductance) plus a shift in voltage-dependence. It has also been shown that 20s of NMDA stimulation, or alternatively, increase of intracellular calcium, increases functional dopamine D1 receptor density at the membrane, which corresponds to an alteration in *κ* for D1 parameters, targeting a number of ion channels simultaneously ^39^. For deep cerebellar neurons, there has recently been some direct evidence on the conditions required that induce intrinsic plasticity. Here, alterations in intrinsic excitability can be induced by bursts of EPSPs and IPSPs, accompanied by dendritic calcium transients ^38^. In striatal MSNs, it has been determined that synaptic stimulation at 1Hz does not cause significant calcium signals, but 10Hz stimulation causes moderate increases, and higher stimulation (up to 100Hz) significantly raises calcium levels ^40^.

In the simulations, neural activation ( *A _n_*) is estimated from the number of spikes generated, measured over the simulated behavior. In the model case, the membrane potential is not used as a separate parameter, because membrane potential and spiking behavior are closely linked. However, when a neuron exhibits prominent upstates (periods of high membrane voltages with a variable number of actual spikes), membrane potential may need to be treated as an additional, independent component of *A _n_*, since a great part of the intracellular calcium signal in striatal MSNs is being generated from high voltage activated NMDA and L-type calcium channels ^41^. The number of spikes produced nonetheless seems important because of the phenomenon of backpropagating spikes. Backpropagating spikes enhance the calcium signal, thus providing a basis for a prominent role for spiking behavior, or firing rate, for defining intracellular calcium. The presence of backpropagation of spikes has recently been confirmed for MSNs ^41^.

In general, the induction of intrinsic plasticity may be linked not only to intracellular calcium. There exists an intricate intracellular system of interactions between diffusible substances like calcium and cAMP, as well as a number of crucial proteins (RGS, calcineurin, PKA, PKC, other kinases and phosphatases) for regulating receptor sensitivity and ion channel properties, which are furthermore influenced by NM receptor activation. Thus the learning parameter *h* may be analyzed as being dependent not only on *A _n_*, but also on [ *NM*], and possibly even a third variable for a–slowly changing–intracellular state.

### Synaptic vs. intrinsic plasticity

Learning by intrinsic excitability seems particularly suitable for striatal MSNs, since they have few lateral connections, which provide only a small part of their total input ^42^. When we have strong recurrent interaction, as in cortex, intrinsic excitability learning needs to adjust activation functions relative to each other, e.g., to ensure optimal distribution of activation functions. This probably happens in the cortical maps, such as frequency maps in auditory cortex ^43^.

In hippocampus, synaptic and intrinsic modulation may potentiate each other (E-S potentiation ^1^), but in other systems (e.g., striatum) antagonistic regulation may also exist (such as LTD combined with positive learning), with effects on the balance of synaptic vs. whole-cell localization for the storage of information.

### Neuromodulation

When ion channels are regulated by neuromodulation, we can use a factor [ *NM*] *κ*, where [ *NM*] is the extracellular concentration of the ligand and *k* the receptor sensitivity (see, Eq. 4). *κ* stands for the influence that a NM signal of a certain strength has on a particular ion channel, i.e., the degree of coupling between NM receptor ligand binding and ion channel modification ^44^. Typically, a signal [ *NM*] will regulate several ion channels in parallel, but there may be different *κ _i_* for each ion channel.

If activation function adaptation proceeds by NM-activated *κ* parameters, rather than unconditioned *μ* parameters, response to stimuli will consist of an early, non-modulated component, where the input pattern is reflected directly in the spiking frequency, and a later, modulated component, where habituation occurs for a learned pattern, or the stored pattern is reflected by overlaying a new stimulus and the stored pattern.

NM signals orchestrate both adjustments in activation function and synaptic input, with NM activation often depressing synapses, but increasing the modulation in the activation function through selected conductance changes (activating *κ*-parameters). As a result, the input component of the signal is reduced in comparison to the stored intrinsic component after NM activation. Presumably, this has a dynamic component, such that for a short time after a strong signal there is an input-dominant phase which is then followed by an intrinsic-dominant phase.

### Homeostasis, permanence and information flow

There are different ideas at the present time what intrinsic plasticity can achieve within a network model of neuronal interaction. Reviews of intrinsic plasticity ^1, 10, 18^ are undecided, whether IP acts mainly to maintain homeostasis, adapting to changes in synaptic strength by keeping neurons within certain ranges but without significant informational capacity, as in the model of ^17^, or whether they are themselves capable of being modified in response to particular patterns of activity in ways that facilitate learning and development ^45^. However, as we have shown, homeostatic adaptation does not exclude information storage under conditions of conditional readout. The synergy between synaptic and intrinsic plasticity may take different forms, beyond E-S potentiation. Based on experimental evidence in different systems ^1, 9, 19^ have listed many possible functions and roles of intrinsic adaptive plasticity.

We have greatly simplified the exposition here by concentrating on spike frequency as a major indicator of neural behavior. Certainly the type of firing (e.g., burst firing) is also under control of neuromodulators, and may be influenced by the distribution and density of ion channels. Single neuron computation is more complex than what can be shown with a single compartment model. In dendritic computation, the coupling of different compartments may be prominently affected by intrinsic plasticity. For instance ^35^, showed a loss of clustering for *K* ^+^ channels on the membrane, induced by high glutamate stimulation, indicating a possible input-dependent regulation of dendritic integration.

Studies of concurrent simulation of synaptic coupling parameters and intrinsic ion channel conductances has concluded that intrinsic and synaptic plasticity can achieve similar effects for network operation ^15^. We have suggested that synaptic and intrinsic plasticity can substitute for each other, and furthermore that this essential functional parallelism could be an indication for *information flow* over time from one modality to the other. The direction of this information flow may be from intrinsic to synaptic for the induction of permanent, morphological changes (such as dendritic spine morphology)–however in some systems (e.g., cerebellum) intrinsic plasticity may also have a permanent component (Purkinje cells) ^5, 46^. The detailed interaction between synaptic and intrinsic plasticity is still an open question. Here we have shown a simple, local learning mechanism for intrinsic plasticity that allows to store pattern information without synaptic plasticity. This is different from theoretical approaches, where activation functions are only being modulated to optimize global measures of information transmission between neurons while the information is exclusively stored in synaptic weights. Further work will be needed to investigate the smooth integration of synaptic and intrinsic plasticity and their respective functions in different systems.

## Conclusions

We wanted to show quantitatively that IP can have significant effects on spike frequency, dependent on the statistical structure of the input. In particular, low correlated input, or input during sensitive (high-voltage membrane) states induces the strongest modulation of spike responses for different activation functions, while highly correlated input acts as drivers for neurons, eliminating subtle differences in the activation function. We suggested that starting from a very general, natural format for a learning rule, which can be biologically motivated, we arrive at simple pattern learning, the basis for feature extraction, and realistic types of neural behavior: population-wide increases/decreases of neural firing rates to novel input stimuli, habituation to known stimuli and history-dependent distortions of individual stimuli. A significant application of this theoretical model exists in the observation of pervasive whole-cell adaptations in selected ion channels ( *I _Na_*, *I _CaL_*) after cocaine sensitization ^47– 49^, with implications of the type of learning that underlies addiction. This would reduce the dynamic range of intrinsic plasticity. Potentially, then, learning in striatum is mediated in part by intrinsic plasticity ^50^, and a reduction in inducible intrinsic plasticity or dynamic range of intrinsic plasticity after cocaine sensitization may contribute to the pathology of addiction.
