# Supplementary material for: Learning intrinsic excitability in medium spiny neurons
Source: F1000Res. 2014 Feb 26;2:88. Originally published 2013 Mar 14. [Version 2] doi: 10.12688/f1000research.2-88.v2 (PMC4264637; doi:10.12688/f1000research.2-88.v2)
Supplement: Peer review history

# Peer review: Referee response for version 2

**DOI:** 10.5256/f1000research.3899.r3890

**Published:** 2014-05-16

**Authors:** Arvind Kumar

**Affiliations:** 1 Neurobiology and Biophysics, University of Freiburg, Freiburg, Germany

While the paper could have been improved by including one of the last suggestions I made in the earlier version, I think at this stage the manuscript is of adequate scientific quality.

I have read this submission. I believe that I have an appropriate level of expertise to confirm that it is of an acceptable scientific standard.

# Peer review: Referee response for version 2

**DOI:** 10.5256/f1000research.3899.r3889

**Published:** 2014-03-31

**Authors:** Fidel Santamaria

**Affiliations:** 1 Department of Biology, University of Texas at San Antonio, San Antonio, TX, USA

I like the paper in its current form. The code is very nicely written and easily available. I suggest to also make it available in the NeuroDB database to reach a wider audience.

I have read this submission. I believe that I have an appropriate level of expertise to confirm that it is of an acceptable scientific standard.

# Peer review: Referee response for version 1

**DOI:** 10.5256/f1000research.353.r3068

**Published:** 2014-01-27

**Authors:** Arvind Kumar

**Affiliations:** 1 Neurobiology and Biophysics, University of Freiburg, Freiburg, Germany

In the first part of the manuscript the author describes the voltage dependent sensitivity of the VI curves of the different ion channels as a function of neuromodulation. This result follows directly from the fact that different ion channels are active at different voltages. Next, the author shows that a plasticity rule can be realised by changing the conductance of different ion channels. The author provides a nice simple demonstration of this interesting idea. But the manuscript needs a major revision to clarify several issues. 

Below are my comments, and I have tried not to repeat the first reviewer. 

I do not think it is correct to refer to the VI curve as the activation function of a neuron. In general it is not correct to use the word ‘variability’ to refer to the modulation of the VI curve due to μ. I think ‘sensitivity’ better captures the observation. 

In Figure 1 the author shows that modulation of the VI curve is maximal around specific voltages. But it seems that this modulation in the VI curve depends on the value of current at that voltage. Obviously, e.g. when I_{Kir} is zero for μ=1 , it does not matter how much you change μ, there will not be any change in the VI curve at that voltage. To make a claim about the sensitivity of the VI curves in different voltage ranges, it should scaled for the VI curve for μ=1. 

The claim that the VI curves can act as basis functions is made in a loose sense and should be strengthened, else remove it from the text. 

Figure 4: Is there a range of μ values for which correlations fail to induce reliable spiking response. In general 0.9 is a very high correlation. How do the neurons behave for more acceptable correlations values e.g. 0.1-0.2?

Induction and maintenance of plasticity: The author wrote “Neural activation is largely determined by intracellular calcium but here we estimate…”. What is the meaning of ‘Neural activation’? Why is it determined by calcium? If it is spike output, then it is mainly determined by the kind of its input.

The learning rule is formulated in such a way that for every input to these neurons, there will be increased response (positive learning) and other way around in the negative learning. So this learning will increase the noise level in the networks. 

The learning rule is unstable, because one neuronal activity is above the threshold θ its firing can only increase. Authors argue that adaption could be a way to stabilise the firing rates, however is not clear whether the model will converge, because if the firing rate drops below θ, then neuron response can only decrease.

Finally, there is another implementation related problem with the learning rule. An external agent is assumed which checks the firing rate of the neuron above or below a baseline firing rate and then updates the ion channel parameters. If we compare it to synaptic plasticity do not require such an agent, because every spike can causes a weight update.

An important question is how the learning rule could switch between negative and positive learning regimes. 

In general, the methods are poorly described. See minor comments for more details. 

**Minor points:**

- Page 1: left col. last para, II line: The sentence does not link with the previous and next sentences. Moreover, it needs to be rephrased; the reference nos. are in superscript, that's why the sentence does not read correctly. 
- I do not understand what the author means by “Thus, intrinsic plasticity is conceptualized as fundamentally different from synaptic plasticity which does not encompass such a state change”. It is obvious that synaptic plasticity can cause state changes. 
- The difference in Eq. 2 and Eq. 3 is not clear with regard to the implementation of the ion channel conductances. What does g_s refer to in Eq. 3? 
- Equation 4:  In the expression [NM]K, it is not clear if ‘K’ is a multiplier to [NM] or subscript. The notations in the Eq. and in the corresponding text do not match. 
- Page 4: EPSP’s should be expressed in the units of voltage and not in terms of the current. As far as I understand this is a point neurone implementation of the MSN, so why should the synaptic strengths be expressed in units of area?
- The method to calibrate the synapses is not clear. What is the meaning of “ …0.6nA, which corresponds to the experimentally measured average value for the rheobase in". At -40mV MSN is already close to its spiking threshold? And rehobase is measured from resting membrane potential? 
- How were the correlations in input generated?
- Figure 1: Please state which variable is plotted on the x- and y-axis. Providing just units is confusing. Also it is not clear which line refers to which μ value? And therefore it is not possible to know in which direct μ affects the v-i curves. I assume that all of the three VI curves are modulated in the same direction. None of the axis measures ‘variability’ in this figure. What is plotted is the change in the VI curve for different values of μ. So you want to refer to this effect as sensitivity of the VI curve.
- Figure 2: Once again I am not sure which axis shows the variability? And axis on the insets are missing. 
- Figure 3: Render the different lines in colours which reflect the voltage. In its current form it's not easy to read these figures.
- Figure 4: the o/p is shown for only one value of i/p firing rate (couldn't find any mention of what value it is). Hence, in addition to the present figure 4, a figure showing i/p firing rate Vs o/p firing rate for more than one data point of i/p firing rate could be included. This can be plotted for all the three values of μ. This on the other hand could be drawn for few values of i/p correlations. 
- Figure 8: It is not clear if the traces are drawn for +ve or -ve learning. The labels of neuron numbers (No. 4,8) are right if it's for negative learning, but should be in reverse order if it's for positive learning.
- Page 9, first paragraph: Update the reference to figures after the changing the figure captions of Fig. 7,8.

I have read this submission. I believe that I have an appropriate level of expertise to confirm that it is of an acceptable scientific standard, however I have significant reservations, as outlined above.

# Response

**Published:** 2014-05-13

**Authors:** Gabriele Scheler

**Affiliations:** Carl Correns Foundation for Mathematical Biology, USA

- *“I do not think it is correct to refer to the VI curve as the activation function of a neuron. In general it is not correct to use the word ‘variability’ to refer to the modulation of the VI curve due to μ. I think ‘sensitivity’ better captures the observation. *
*In Figure 1 the author shows that modulation of the VI curve is maximal around specific voltages. But it seems that this modulation in the VI curve depends on the value of current at that voltage. Obviously, e.g. when I_{Kir} is zero for μ=1 , it does not matter how much you change μ, there will not be any change in the VI curve at that voltage. To make a claim about the sensitivity of the VI curves in different voltage ranges, it should scaled for the VI curve for μ=1.”*
  Have added the variable names to the axes, as well as the μ values.
  “Variability” has been replaced with “modulation” throughout the text.
  Activation function has been replaced by I-V curve.
- *“The claim that the VI curves can act as basis functions is made in a loose sense and should be strengthened, else remove it from the text.”*
  This has been removed.
- *“Figure 4: Is there a range of μ values for which correlations fail to induce reliable spiking response. In general 0.9 is a very high correlation. How do the neurons behave for more acceptable correlations values e.g. 0.1-0.2?”*
  These extreme values have been used for demonstration in this figure.
- *“The learning rule is formulated in such a way that for every input to these neurons, there will be increased response (positive learning) and other way around in the negative learning. So this learning will increase the noise level in the networks. *
*The learning rule is unstable, because one neuronal activity is above the threshold θ its firing can only increase. Authors argue that adaption could be a way to stabilise the firing rates, however is not clear whether the model will converge, because if the firing rate drops below *θ, *then neuron response can only decrease.”*
  This is not guaranteed to be stable; additional mechanisms (additional research), for example synaptic plasticity in the other direction, may be needed.
- *Finally, there is another implementation related problem with the learning rule. An external agent is assumed which checks the firing rate of the neuron above or below a baseline firing rate and then updates the ion channel parameters. If we compare it to synaptic plasticity do not require such an agent, because every spike can causes a weight update.”*
  Have added a clarifying sentence.
- *“An important question is how the learning rule could switch between negative and positive learning regimes.”*
  The negative/positive learning rule can depend on each neuron. We did not investigate this for networks of neurons.

**Minor Comments:**

- *“I do not understand what the author means by “Thus, intrinsic plasticity is conceptualized as fundamentally different from synaptic plasticity which does not encompass such a state change”. It is obvious that synaptic plasticity can cause state changes.”*
  Have added a clarifying sentence.
- *“The difference in Eq. 2 and Eq. 3 is not clear with regard to the implementation of the ion channel conductances. What does g_s refer to in Eq. 3?”*
  There was a typo in Eq. 3, which has now been fixed (please see response to Fidel Santamaria).
- *“Equation 4:  In the expression [NM]K, it is not clear if ‘K’ is a multiplier to [NM] or subscript. The notations in the Eq. and in the corresponding text do not match.”*
  This was an error introduced during typesetting; it has been corrected to κ (lowercase kappa).
- *“Page 4: EPSP’s should be expressed in the units of voltage and not in terms of the current. As far as I understand this is a point neurone implementation of the MSN, so why should the synaptic strengths be expressed in units of area?”*
  All units in the simulation are expressed in area units; V _EPSP_ = 1/g _s_ * I _EPSP_
- *“How were the correlations in input generated?”*
  Referred to code.
- *“Figure 1: Please state which variable is plotted on the x- and y-axis. Providing just units is confusing. Also it is not clear which line refers to which μ value? And therefore it is not possible to know in which direct μ affects the v-i curves. I assume that all of the three VI curves are modulated in the same direction. None of the axis measures ‘variability’ in this figure. What is plotted is the change in the VI curve for different values of μ. So you want to refer to this effect as sensitivity of the VI curve.”*
  Added plotted variables at the axes, and added μ values.
- *“Figure 2: Once again I am not sure which axis shows the variability? And axis on the insets are missing.”*
  Added plotted variables at the axes, and added μ values.
- *“Figure 3: Render the different lines in colours which reflect the voltage. In its current form it's not easy to read these figures.”*
  This has been done.
- *“Figure 4: the o/p is shown for only one value of i/p firing rate (couldn't find any mention of what value it is). Hence, in addition to the present figure 4, a figure showing i/p firing rate Vs o/p firing rate for more than one data point of i/p firing rate could be included. This can be plotted for all the three values of μ. This on the other hand could be drawn for few values of i/p correlations.”*
  One could have a figure with correlation on the x axis, and firing rate on the y. For low correlations all three neurons are at 20, 26, 40. At a certain, higher, correlation rate it switches from these frequencies to reliable firing modes correlated with the input – there might be different correlation values for different neurons.
- *“Figure 8: It is not clear if the traces are drawn for +ve or -ve learning. The labels of neuron numbers (No. 4,8) are right if it's for negative learning, but should be in reverse order if it's for positive learning.”*
  It is negative learning, consistent with the original caption.
- *“Page 9, first paragraph: Update the reference to figures after the changing the figure captions of Fig. 7,8.”*
  This has been done.

# Peer review: Referee response for version 1

**DOI:** 10.5256/f1000research.353.r936

**Published:** 2013-05-09

**Authors:** Fidel Santamaria

**Affiliations:** 1 Department of Biology, University of Texas at San Antonio, San Antonio, TX, USA

This is an interesting paper that aims to elucidate the contribution of intrinsic excitability plasticity on neuronal learning using computer models. 

The paper would benefit from presenting a statistical analysis of the learning process. What is the probability of distinguishing a learnt pattern as opposed of detecting a false positive?

The fact that P _noise_ also evokes a close firing rate response as P _learn_ should be closely addressed. Is the output for P _noise_ and P _learn_ significantly different? There should be controls for having the same number of input neurons to any pattern. Otherwise, the results in Figure 6 could be due to the overlap of P _noise_ and P _learn_. Does the author propose a metric by which the neurons distinguish the learnt from the noisy pattern?

Figure 7 and 8 are mixed up in the text and with their captions. Please re-arrange. In the histogram of what is now Fig. 7 the author again shows that P _noise_ is capable of eliciting a response that seems to be different from background. Is this significantly different (statistically speaking)? 

I think there’s a typo in Eq. 3.

Although the premise of the paper is quite interesting I think the results and discussions would be much better supported with a thorough statistical analysis of the spiking response of the model.

I have read this submission. I believe that I have an appropriate level of expertise to confirm that it is of an acceptable scientific standard, however I have significant reservations, as outlined above.

# Response

**Published:** 2014-05-13

**Authors:** Gabriele Scheler

**Affiliations:** Carl Correns Foundation for Mathematical Biology, USA

- *“Figure 7 and 8 are mixed up in the text and with their captions. Please re-arrange.”*
  This was a mixup during publication, and has now been corrected.
- *“I think there’s a typo in Eq. 3”*
  This has been corrected, so it reads: [μ _1_ *g*_1_(V _m_-V _0_) + .... + *g*_s_(V _m_-V _0_)]
